# Intratumoral injection and retention hold promise to improve cytokine therapies for cancer

**DOI:** 10.3389/fonc.2024.1456658

**Published:** 2024-08-26

**Authors:** Karsten Sauer, Kavya Rakhra, Kaida Wu, Naveen K. Mehta, Jennifer S. Michaelson, Patrick A. Baeuerle

**Affiliations:** ^1^ Cullinan Therapeutics, Cambridge, MA, United States; ^2^ Institute of Immunology, Ludwig Maximilians Universitaet Muenchen, Planegg, Germany

**Keywords:** cytokine, intratumoral, IL-2, IL-12, collagen, CLN-617, immunotherapy, cancer

## Abstract

As powerful activators of the immune system, cytokines have been extensively explored for treating various cancers. But despite encouraging advances and some drug approvals, the broad adoption of cytokine therapies in the clinic has been limited by low response rates and sometimes severe toxicities. This in part reflects an inefficient biodistribution to tumors or a pleiotropic action on bystander cells and tissues. Here, we first review these issues and then argue for the intratumoral delivery of engineered cytokine fusion proteins that have been optimized for tumor retention as a potential solution to overcome these limitations and realize the potential of cytokines as highly effective therapeutics for cancer.

## Introduction

In this perspective, we advocate for the intratumoral (i.t.) injection of engineered cytokine molecules with optimized tumor retention as a potential solution to overcome the limitations which have thus far hindered the broad adoption of cytokines as safe and effective cancer immuno-therapeutics. The promise of this strategy is increasingly being recognized (1, 2).

As soluble and potent immune activators, cytokines play key roles in orchestrating productive anti-tumor immune responses (3). For this reason, several cytokines have been explored as cancer immunotherapeutics. The currently most pursued ones include interleukin 2 (IL-2), IL-12, IL-15, IL-18, interferons (IFNα, β and γ), tumor necrosis factor α (TNFα), and granulocyte-monocyte colony-stimulating factor (GM-CSF). But despite encouraging examples of efficacy, the clinical use of cytokines is relatively rare, and approved products are limited to IL-2 (Proleukin), IFNα (Besremi, Pegasys, PegIntron, Intron A) and GM-CSF (Leukine). Additionally approved viral products include the IFNα-2b expressing adenovirus Nadofaragene firadenovec-vncg (Adstiladrin), and the GM-CSF expressing oncolytic virus (OV) Talimogene laherparepvec (T-VEC, product name Imlygic). However, these products have only been approved in a narrow set of indications (4–6).

The paucity of approved cytokine products may reflect dose-limiting toxicities (DLT) and low response rates of systemically administered cytokines (2, 3, 5). The toxicity of pro-inflammatory cytokines primarily comes from their pleiotropic action on bystander cells, along with a fundamental difference in how endogenous and exogenously administered cytokines are regulated. Endogenous cytokines are produced locally at sites of inflammation, act in an auto- or paracrine fashion and are quickly consumed by their target cells. This limits systemic cytokine exposure, which if dysregulated can cause severe toxicities such as cytokine release syndrome (CRS) - prominently seen in COVID-19 patients (7, 8). For cancer therapy, cytokines are typically administered systemically at high doses and repeatedly to ensure sustained engagement of the targeted immune cells in tumors. This will however expose unintended target cells and tissues expressing the respective cytokine receptor, causing toxicities (2). A well-documented example are the vascular leak syndrome (VLS) and pulmonary edema caused by IL-2 binding to receptors on lung endothelial cells (2, 5, 9).

Low cytokine efficacy can be caused by short serum half-lives and an inefficient biodistribution to tumors and tumor-draining lymph nodes (tdLN). Moreover, wildtype IL-2 can engage both desired effector T cells and NK cells, and undesired immune-suppressive T_reg_ cells (5, 10). These factors limit the activation of immune cells by the administered cytokine, particularly when given at sub-efficacious concentrations due to low maximum tolerated doses (MTD). Moreover, feedback-inhibition (tachyphylaxis) can limit the efficacy of repeatedly administered cytokines such as IL-12 (11).

## Limitations of current cytokine modalities

Multiple approaches have been explored to improve the safety and efficacy of cytokine therapeutics for cancer. Broadly, these can be categorized as systemically delivered modalities or as modalities that are i.t.-injected directly into their desired sites of action.

### Systemically delivered cytokines

Since this perspective focuses on i.t. administered cytokines, we only briefly discuss systemically administered modalities here and refer to excellent recent literature for more details (2, 3, 5, 12–15). Advantages of systemic cytokine delivery include simple administration and predictable pharmacokinetics (PK) in serum. Recent approaches for systemic delivery aim to minimize toxic “off-tumor” activity, increase activity within tumors and prolong cytokine exposure. Methods include (i) altering cytokine specificity for receptor subunits, (ii) engineering cytokines for increased stability, (iii) masking cytokines in circulation, (iv) fusing cytokines with tumor- or effector cell-targeting moieties, (v) embedding cytokines in biomaterials which accumulate in tumors, and (vi) expressing cytokines only within tumors (10).

All these approaches have distinct advantages and limitations, which may explain their limited success in the clinic to date. Receptor-biased cytokine ‘muteins’ are designed for reduced binding to target cells mediating toxicities or tachyphylaxis. For example, so-called non-alpha IL-2 variants avoid binding to the high-affinity IL-2R α-subunit (CD25) expressed on lung epithelia, NK cells and T_reg_ cells (3). However, CD25 is also expressed and upregulated on activated T cells and important for effector responses and IL-2 synergy with PD-1 blockade (16). The lack of CD25 binding might explain why neither non-alpha muteins nor similar IL-15 variants have succeeded in patients yet (16). Likewise, cytokines fused to albumin, immunoglobulin Fc domains or polyethylene glycol (PEG) polymers for half-life extension have not yet borne out in the clinic. This might reflect limited tumor penetration, toxicities or, possibly, exacerbated tachyphylaxis due to prolonged systemic exposure of the cytokine. Similar concerns apply to masked cytokine prodrugs that are activated by tumor-resident proteases, ATP or the low intratumoral pH. Here, heterogeneous or insufficient presence of the activating mechanisms in tumors or tdLN may limit efficacy, and drainage of the activated cytokine from tumors might limit efficacy or cause toxicities (2, 5).

So-called immuno-cytokines and other modalities incorporating tumor-targeting moieties are designed to enrich cytokines in tumors while limiting systemic exposure. However, much of the biodistribution is governed by binding of the cytokine moiety to its receptors on peripheral immune cells rather than tumor cells. This causes cytokine-related toxicities and limits tumor exposure (10). An alternative strategy is to selectively deliver cytokines *in cis* to targeted immune cells (e.g., CD8 T cells) in the periphery via immuno-cytokines or cytokine-releasing nanoparticles. Although effector cells are targeted in the periphery in this case, the cytokines are expected to be maximally active only in the tumor and tdLNs, where the respective high-affinity cytokine receptors are preferentially upregulated. Whether these approaches, or the adoptive transfer of tumor-specific T cells loaded with immuno-cytokines or cytokine-releasing nanoparticles, increase the so far limited success of immuno-cytokines in cancer patients remains to be shown (2, 15, 17). The conceptually related infusion of tumor-specific T cells engineered to express cytokine genes may be limited by toxicities due to variable cytokine expression and short durability of engraftment, and by the high cost and challenging logistics of engineered cell therapies (18, 19).

Finally, the systemic administration of OVs engineered to express cytokines such as GM-CSF has so far been safe in patients but elicited lower response rates than i.t. delivery. This approach is further challenged by complicated logistics and biosafety considerations, uncertainty about how much cytokines versus direct tumor cell lysis contribute to efficacy, unclear optimal doses, and a need for better understanding of PK and neutralization by anti-OV immune responses (4). Altogether, even advanced modalities have not yet led to a broad clinical success of systemically delivered cytokines.

### Intra-tumorally delivered cytokines

**Figure 1:**
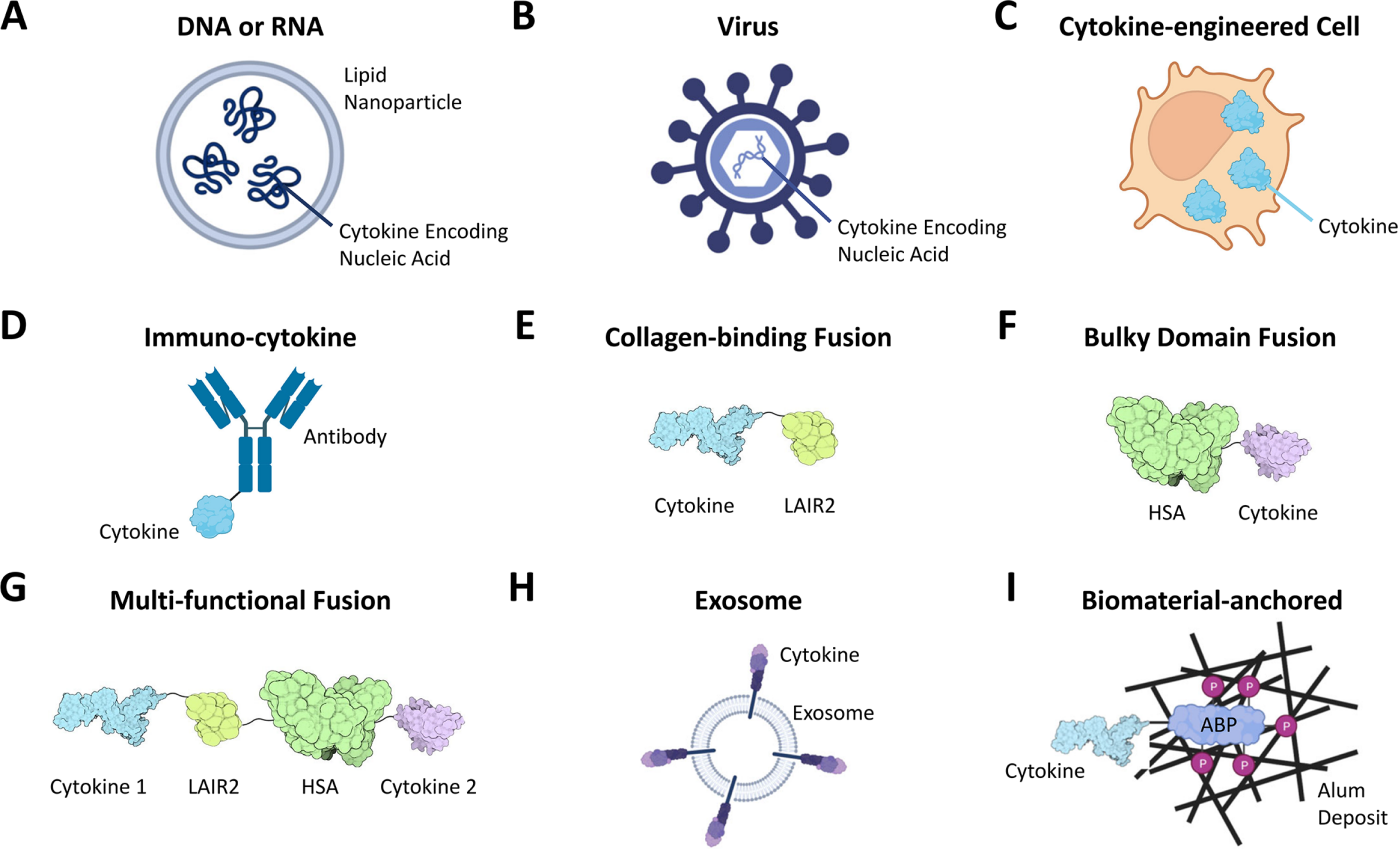
Cytokine modalities for i.t. administration include free or lipid nanoparticle-loaded DNAs or RNAs encoding cytokines **(A)**, cytokine expressing OV or other viruses **(B)**, and cells engineered to express exogenous cytokines **(C)**. Additional modalities engineered for cytokine stabilization and retention at sites of injection include immuno-cytokines containing both cytokines and tumor antigen-binding moieties (typically an antibody, **D**), cytokine fusions with tumor matrix/collagen-binding domains such as LAIR2 **(E)** or with bulky domains such as human serum albumin (HSA, **F**) or multi-functional fusions of several cytokines with both collagen-binding and bulky domains (**G**, ref. 43), liposomes or exosomes “presenting” cytokines on their surface **(H)** and cytokines anchored to exogenous biomaterial deposits in tumors (**I**, shown is a cytokine fused to a phosphorylated alum-binding peptide [ABP] whose phosphate residues [P] undergo ligand-exchange reactions with alum deposits (49)). For details, see text. Created with BioRender.com.

A conceptually attractive alternative approach to maximize “on-tumor” exposure and minimize “off-tumor” systemic exposure is to directly inject cytokine therapeutics into tumors. Initially, i.t. delivery was limited to easily accessible body surface-located tumors such as melanoma, but advances in image-guided delivery and robotic endoscopy now allow treating lesions deeper in the body. Many more cancer indications can now be addressed, including breast, lung, head and neck, cervical, pancreatic, prostate, colorectal, liver, ovarian and kidney cancer, sarcoma and glioblastoma (**Table 1**, **Supplementary Table 1**) (1, 10, 46–48). Modalities for i.t. cytokine delivery include cytokine-encoding mRNAs or DNAs alone or contained in lipid nanoparticles (LNP), OVs or other viruses encoding cytokines, cytokine-expressing transgenic cells, immuno-cytokines, recombinant cytokines and biomaterial-anchored cytokines (**Figure 1**) (1, 2, 5, 46, 48–52).

**Table 1:** I.t. injected cytokines that have reached clinical trials.

Modality	Asset	Cytokine(s)	Phase	Indication(s)	Comments*	References
OV	T-VEC (Talimogene laherparepvec, Imlygic/Amgen)	GM-CSF	2 & 3	Melanoma	- *First approved OV and IT asset* - Single agent activity with ORR up to 31.5%, higher in sub-patient populations - Low response rate in patients with visceral metastases - ORR 39% in combo with ipilimumab - ORR 42-67% in combo with pembrolizumab, 3 year OS 71% - All treatment well tolerated	(5, 20–22) *(Reviews of multiple studies)*
	OrienX010 (OrienGene Biotechnology)	GM-CSF (±αPD-1)	1/2	Melanoma	- ORR generally ≤28.6% - ORR 20.7% in combo with toripalimab for stage IV (M1c) liver metastases - Showed abscopal effects - Treatment tolerated	(22–25)
	VG2025 (Virogin Biotech)	IL-12 + IL-15	1	Solid Tumors	- ORR 25% (n=4) - No DLT, acceptable safety	(22, 26)
cDNA Plasmid	Tavokinogene telseplasmid (TAVO, Oncosec Medical/Merck)	IL-12	2 (+ Pembrolizumab or Nivolumab)	Melanoma, TNBC	- Pembro combo showed ORR 10.2% (did not meet ORR endpoint in PD-1 refractory melanoma); also showed ORR 41% in advanced melanoma with low PD-1^high^ CTLA-4^high^ CD8^+^ CTL (n=22) - Neoadjuvant combo with Nivolumab: Pre-operative response rate 77.8% (n=9) - No tumor retention of the drug - Showed systemic immune responses - Well tolerated	(22, 27–29)
Tumor-matrix binding immuno-cytokine	Mixture of L19−IL2 + L19−TNFα (Nidlegy/Daromun, Philogen)	IL-2 + TNFα (Fibronectin ED-B domain-targeted)	2/3 (pivotal)	Melanoma, skin cancers	*NCT02938299 Ph 3:* - Neoadjuvant Nidlegy + surgery improved relapse-free survival (RFS) vs. surgery alone (HR = 0.59) - Median RFS 16.7 vs. 6.9 months - 21% complete pathological responses - Manageable TRAE *NCT02076633 Ph 2, completed:* - In melanoma, ORR 50% (week 12) - Robust abscopal effects - Well tolerated	(21, 22, 30–32)
	L19−IL2 (Darleukin, Philogen)	IL-2 (Fibronectin ED-B domain-targeted)	2	Melanoma	- Stage IIIB/IIIC (n=24): ORR 53.9%, CR 25% (6/24, 5 patients with DOR >24 months) - Median survival 905 days - Well tolerated - Decreased MDSC and transiently increased CD4 T_reg_ cell proportions in blood samples. Transiently increased total NK cells and CD8 T cells in blood.	(22, 33) and **Table 2**
Immuno-cytokine	Hu14.18-IL2/(APN301/EMD273063, EMD/Apeiron Biologics/Lexigen)	IL-2 (GD2-targeted)	2	Melanoma, neuroblastoma	- *Completed* - mOS in resectable stage III/IV melanoma 61.6 months (18 patients) - Reversible toxicities - TIL observed in on treatment biopsy associated with efficacy	(22, 34–37) and **Table 2**
Recombinant or natural cytokine	Proleukin	IL-2	2	Melanoma	- *Completed* - Favorable 2 year OS (95.5% stage IIIB, 72% IIIC, 66.7% IV M1a) - Well tolerated	(38) and **Table 2**
	IL-2	IL-2		Melanoma	- *Comparison of 5 studies in 2001-2011* - Variable ORR of 25% to 99.5% - Well tolerated	(39) and therein
	Natural IFNα or recombinant IFNα 2b	IFNα		Melanoma	- ORR 18% (9/51)	(40)
	Recombinant IFNα	IFNα	2	Melanoma	- ORR 25% - Well tolerated	(41)
	Recombinant IFNα 2b	IFNα	2	Melanoma	- ORR 14.3%	(42)
Tumor-retained IL-2-IL-12 fusion protein	CLN-617 (Cullinan Therapeutics)	IL-2 + IL-12	1	Solid tumors	- *Clinical study initiated Q4, 2023*	(43, 44)
Cytokine anchored to exogenous biomaterial deposits	ANK-101	IL-12	1	Solid tumors	- *Clinical study initiated Q1, 2024*	(2, 22, 45)

Listed here are clinical i.t. cytokines discussed in the text. Additional examples are listed in **Supplementary Table 1**. *Monotherapy unless indicated otherwise. Chemo, chemotherapy; CPI, checkpoint inhibitor/blocker; CR, complete response; CTCL, cutaneous T-cell lymphoma; CTG, ClinicalTrials.gov; DLT, dose-limiting toxicity; DOR, duration of response; GBM, glioblastoma; HR, hazard ratio; MKI, multi-kinase inhibitor; mOS, median OS; MSC, mesenchymal stem cell; MSS, microsatellite-stable; MTD, maximum tolerated dose; NMIBC, Non-Muscle Invasive Bladder Cancer; ORR, overall response rate; OS, overall survival; P/C, pemetrexed/cisplatin; Pembro, pembrolizumab; PR, partial response; RFS, relapse-free survival; RT, radiation therapy; SAE, serious adverse events; SD, stable disease; SOC, standard of care; TRAE, treatment-related adverse effects.

Continued in **Supplementary Table 1**.

In comparing key features, i.t. delivered modalities fare favorably over systemically administered modalities, in particular when the i.t. modalities are engineered for tumor retention (1, 5, 10). Moreover, i.t. injection of exogenous cytokines mimics the local production and auto-/paracrine mode of action of endogenous cytokines in diseased tissues with its associated advantages. In particular, the high cytokine doses in tumors achieved by i.t. injection enable saturated receptor occupancy, followed by a slow and reduced systemic distribution - conditions that are generally unachievable with systemically delivered cytokines at the MTD. The improved control of exposure and PK in tumors can allow efficacious dosing without major systemic toxicity. Importantly, i.t. delivery provides cytokines immediate access to tertiary lymphoid structures (TLS) in tumors and to tdLNs, both important sites for initiation, priming and maintenance of anti-tumor T cell immunity (5). This is critical because the therapeutic efficacy of i.t. administered cytokines relies on an abscopal effect, where local injection into one or a few tumors triggers a systemic anti-tumor immune response that eliminates non-injected tumors as well. Rather than systemic cytokine exposure, the mechanism involves T cell priming and activation in tumors and tdLNs (1, 53).

A considerable number of clinical trials have investigated i.t. administered cytokine modalities [**Tables 1**, **2**, **Supplementary Table 1** and (1, 48)]. Most are still ongoing, but several have reported initial results. I.t- delivered cytokines are generally well tolerated. Several examples of significant single-agent efficacy in solid tumor indications have been reported. They include overall response rates (ORR) of up to 31.4% for the GM-CSF expressing OV Imlygic (Talimogene laherparepvec/T-VEC, the first approved OV and i.t. modality) (5, 20, 21), 28.6% for the GM-CSF expressing OV OrienX010 (23–25), and 25% for the OV VG2025 expressing both IL-12 and IL-15 (26). Other examples include ORRs of 50% for the tumor matrix-binding IL-2 + TNFα immuno-cytokine mix Nidlegy/Daromun (which also improved recurrence-free survival for patients with locally advanced fully resectable melanoma) or single-agent matrix-binding IL-2 Darleukin (which also yielded a median survival of 905 days) (21, 30–33, 48), a median OS of 61.57 months for the GD2-targeted immuno-cytokine Hu14.18-IL2 which exceeded that achieved by i.v. administration (34–37), variable ORRs of 25-99.5% for i.t. injected IL-2, and ORR of up to 25% for recombinant IFNα. There are multiple reports of abscopal effects or other evidence of systemic immune activation. Efficacy is often increased by combination with checkpoint blockade or other treatments (**Tables 1**, **2**, **Supplementary Table 1**). Notably, these reports of efficacy of i.t. modalities extend beyond OVs – where the cytokine may function together with other viral mechanisms of action – to include tumor-targeted immuno-cytokines and naked cytokine proteins, thus far primarily focusing on IL-2 and IFNα. However, many more drug candidates are under investigation (5, 21, 48).

**Table 2:** Juxtaposition of clinical data for i.t. versus i.v. administered IL-2 modalities.

Modality	Asset	Indication	Route	Application Schedule & Dose	Pharmacodynamics	Toxicity Profile	Efficacy	References
Recombinant human IL-2	Proleukin	Melanoma	i.v.	- Every 8 hours for up to 14 consecutive doses over 5 days - 600,000 or 720,000 IU/kg - Second identical treatment cycle scheduled after 6 to 9 days of rest - courses could be repeated every 6-12 weeks	- Systemic IL-2 can cause transient increases in CD4 T_reg_ cells	- Severe toxicities, reversed after treatment termination - 6 patients (2%) died from adverse events, all related to sepsis	- *8 clinical trials, n=270* - ORR 16% - 6% CR - >50% of CR progression-free after 5 years - No progression in patients who had responded for >30 months.	(54, 55)
			i.t.	- 3 x weekly, individually escalated doses - Median duration 6.5 weeks - Median total IL-2 dose 72 million IU - Median 10 injected metastases	- A dose-dependent inflammatory reaction at site of injection induced selective necrosis of tumor tissue associated with an intra- and peritumorous lymphocytic infiltrate mainly of CD3^+^ T cells and some CD3^-^CD56^+^ NK cells	- Well tolerated - Adverse events mainly grade 1-2 - Most common: local erythema and slight local swelling	- n=72 - 25% recurrence-free - Up to 11 years of follow-up - Favorable 2 year OS (95.5% stage IIIB, 72% IIIC, 66.7% IV M1a) - 36.7% response rate to subsequent chemotherapy	(38, 56)
				- Biweekly with goal of 4 sessions - Mean 5 sessions - Average dose 10.4 million IU		- Well tolerated - Minor discomfort - 85% flu-like symptoms, resolved in 24-48 hr	- n=39 - ORR 82% - 51% CR (eventually relapsed in 20%), 31% PR - 80% 5-year survival of CRs, 33% of PRs	(39)
				- Twice weekly - 3-18 million IU		- Well tolerated - Only few mild side effects (Grade 1-2)	- n=7 - ORR 99.5% - 96% CR, 3.5% PR	(57)
IL-2 Immuno-cytokine	L19−IL2 (Darleukin, Philogen)	Melanoma, stage IIIB/IIIC	i.t.	- Weekly for 4 weeks - Maximum dose 10 million IU	- In 76% of patients, inflammatory injection site reaction limited to tumor tissue, followed by selective tumor necrosis - Decreased MDSC and transiently increased CD4 T_reg_ cell proportions in blood samples - Transiently increased total NK cells and CD8 T cells in blood - Sustained increase in frequency and absolute count of lymphocytes (mainly CD4 T cells)	- Well tolerated - Mostly grade 1-2 toxicities - No SAE	- n=24 - ORR 53.9%, CR 25% - 5 patients with DOR >24 months - Median survival 905 days	(22, 33)
	Hu14.18-IL2 (APN301/EMD273063, EMD/Apeiron Biologics/Lexigen)	Melanoma, neuroblastoma	i.t.	- 3 courses of 6 mg/m^2^/day on days 1, 2 and 3 of each 28-day course - Corresponds to about 18 million IU/m^2^/day of IL-2 - Treatment was neoadjuvant (Group A) or post resection (Group B)	- TIL and immune signatures in on treatment biopsy associated with efficacy in Group A (Neoadjuvant) - All 18 patients developed anti-drug antibodies - Treatment induced transient lymphopenia on day 3 with subsequent rebound lymphocytosis - Increased levels of soluble IL-2Rα and CRP suggesting immune activation	- Reversible and manageable toxicities, including IL-2 constitutional symptoms, grade 1-2 laboratory changes, hypotension and pain - Dose reductions required for several patients - MTD 7.5 mg/m^2^/day (Phase 1 trial)	- n=18 - mOS in resectable stage III/IV melanoma 61.6 months - No difference by GD2 status	(22, 34–37)
		Melanoma	i.v.	- 6 mg/m^2^/day on days 1, 2 and 3 of each 28-day cycle - Corresponds to about 18 million IU/m^2^/day of IL-2 - 2-4 cycles	- Peripheral blood lymphopenia on day 3 followed by lymphocytosis on Day 8 and increased CRP - Transiently increased serum sIL-2Rα - No correlation between peak drug level on Day 1 and toxicity or response - 13 patients developed anti-drug antibodies (93%)	- Reversible toxicities, including grade 3 thrombocytopenia and blood chemistry, and one transient grade 4 lymphopenia - Grade 3 hypotension (n=2) and grade 2 renal insufficiency (n=1) required dose reductions in 3 patients who had a PR or SD	- n=14 - 1 transient PR (7.1%)	(58)
		Neuroblastoma		- 12 mg/m^2^/day for 3 days every 28 days - Corresponds to about 36 million IU/m^2^/day of IL-2	- Multi-modal mechanism of action where Fc-portion mediates ADCC and CDC while IL-2 moiety activates NK cells and T cells - hu14.18-IL2 peak serum levels similar for responders and nonresponders - Transient lymphopenia followed by lymphocytosis consistent with immune activation - Transiently increased serum sIL2R, no association with DLT - 13-16 patients developed anti-drug antibodies, not associated with drug serum levels or responses - No association between factors at diagnosis and responses	- n=38 - Phase 2 DLT included vascular leak, hypotension, hypoxia, pain, allergic reactions, transaminitis, hyperbilirubinemia - Most toxicities reversible	- No responses in measurable/bulky disease (n=13) - 21.7% CR with 9 to >35 month durability in patients with non-measurable disease (n=23) - Overall 63% 1-year OS	(59)

A comparison of clinical data for IL-2 modalities that have been well studied in both i.v. and i.t. administration settings indicates that either mode of administration can elicit significant anti-tumor efficacy at overlapping doses (**Table 2**). However, efficacy tended to be higher after i.t. administration, even at lower doses. Moreover, i.v. administration caused severe toxicities which were not seen upon i.t. administration of the same modality, which was usually well tolerated. This holds true for recombinant human IL-2 (Proleukin) as well as for immunocytokines including Hu14.18-IL2. Although there are caveats due to differences in the precise doses, treatment regimen, trial designs and patient populations between the different studies, these findings do point to notable advantages of i.t. administration.

Among modalities, i.t.-injected or electroporated cytokine-encoding cDNAs, mRNAs and viruses have in particular been widely explored in clinical trials (**Figures 1A, B**; **Table 1**, **Supplementary Table 1**). Here, transfected or transduced cells in the tumor produce the cytokine and other payloads. OVs preferentially propagate in and kill tumor cells through additional mechanisms. The promise of localized cytokine production in tumors, at least when combined with other OV mechanisms, is illustrated by the safety and efficacy of the OVs discussed above, and by the FDA approval of Imlygic. Yet, complex biosafety requirements and logistics limit OV application, and efficacy upon injection into large tumors can be limited to areas near the needle track, as seen for the TNF-producing virus TNFerade (4, 5, 48, 50, 60). Highlighting another limitation, infrequent responses of visceral metastases in patients indicate an insufficient abscopal effect of i.t. administered Imlygic in Phase 3 studies (20).

While usually safe, the clinical efficacy of non-OV DNA and RNA modalities so far has been variable, being sometimes significant but in other cases not, particularly as a monotherapy (**Table 1**, **Supplementary Table 1**) (2, 21, 48). This likely reflects difficulties in achieving consistent expression inside injected tumors, reaching effective cytokine doses and sufficiently controlled exposure, along with cytokine leakage out of tumors and peripheral turnover. Remarkably, the four cytokine-encoding mRNA combination BNT131/SAR441000 had low efficacy and its trial was discontinued (61–63). Tavokinogene telseplasmid (TAVO) did not meet its ORR endpoint in PD-1 refractory melanoma when combined with pembrolizumab but has yielded significant response rates in checkpoint combinations in certain patient populations (**Table 1**) (27, 28). Clearly, approaches employing nucleic acids and particularly OVs have promise, but more work is needed to optimize them for indirect cytokine delivery. The same is true for i.t. injection of cytokine gene-engineered cells (**Figure 1C**), which so far had limited efficacy in initial trials (5).

Altogether, notwithstanding some promising examples of efficacy, multiple trials of i.t.-delivered cytokine therapeutics reported little or no efficacy and have been terminated or discontinued (**Supplementary Table 1**). The reasons likely depend on the modality, mechanism of action, and the specific design and/or patient population studied. One major problem is that i.t. injected or intratumorally produced cytokines can quickly diffuse into circulation, in particular, when initial i.t. concentrations are high and saturate binding sites within tumors, or when cytokine release rates exceed rates of uptake by target cells in tumors. This leakage from injected tumors then causes systemic exposure to the cytokine and greater than expected toxicities (2, 10, 48, 64, 65).

## Promising new developments

One solution to avoid leakage from tumors is to endow i.t.-delivered cytokines with moieties that anchor them to the tumor microenvironment or limit diffusion out of tumors. This has been achieved in various ways. For example, cytokine retention in tumors can be achieved via fusion to antibodies specific for tumor antigens (**Figure 1D**). However, downregulation of a targeted tumor antigen on therapy or its heterogeneous expression on tumors could diminish tumor retention of immuno-cytokines. Nevertheless, the clinical safety and efficacy of the immuno-cytokines Daromun, Darleukin and Hu14.18-IL2 highlight the promise of designed tumor-retention following i.t.-delivery. Retention of cytokines in the tumor microenvironment may also enable the delivery of cytokine combinations that would otherwise be intractable.

Cytokine retention in tumors can also be achieved by targeting collagen, an abundant component of nearly all tumors, via fusion to collagen-binding proteins such as lumican or LAIR2, or via fusion to bulky moieties such as human serum albumin (HSA) (**Figures 1E, F**) (2, 10, 66, 67). One example is CLN-617, a fully human fusion protein comprising IL-2, IL-12, LAIR2 and HSA (**Figure 1G**) (43). To our knowledge, CLN-617 is the first clinical modality that co-delivers IL-2 and IL-12 on a single molecule. It builds on the promising safety and efficacy observed with i.t. co-administered collagen-binding IL-2 and IL-12 combined with radiation therapy in spontaneous canine metastatic melanoma (68). To enhance tumor retention, CLN-617 leverages both LAIR2 and HSA. Employing LAIR2 for collagen-binding has two advantages: First, its ability to bind multiple types of collagen may mitigate potential challenges due to heterogeneous collagen expression among tumors and metastases (69). Second, LAIR2 might block immune-inhibition by the immune cell-expressed “checkpoint” receptor LAIR1, which binds collagen with lower affinity (70). Delivery via i.t. injection limits potentially toxic retention in collagen-rich normal kidney or liver tissues. The HSA moiety provides a mechanistically distinct, complementary means of tumor retention: reduced diffusion of bulky payloads out of tumors (2, 10, 66, 67). CLN-617 has additional beneficial properties such as encoding wildtype cytokines, and by co-delivering IL-2 and IL-12, mimicking a natural immune response where multiple cytokines typically act in concert in a local manner. This is exemplified by the known synergy of IL-2 and IL-12 in enhancing T cell and NK cell responses and anti-tumor immunity through mechanisms which include mutual receptor-upregulation (43). An i.t.-delivered murine surrogate of CLN-617 exhibited compelling and safe single-agent anti-tumor efficacy dependent on its retention in tumor tissue, strong abscopal effects and over 10-fold higher tumor than systemic exposure in preclinical models. It also synergized with systemically delivered PD-1 blockade (43). CLN-617 is currently in a Phase I clinical trial both as a monotherapy and in combination with PD-1 blockade (NCT06035744).

Another approach for prolonging tumor-retention is embedding cytokines on the surface of liposomes or exosomes before i.t. injection (**Figure 1H**) (2, 10, 51, 66). However, cytokine-containing liposomes are compromised by rapid endocytic clearance or biodegradation, and encapsulation in hydrogels or chitosan minimizes cytokine bioavailability (10). It remains to be investigated whether such issues contribute to the so far limited clinical efficacy of IL-12 displaying exosomes (CDK-003/ExoIL-12) and mRNA lipid nanoparticles (LNP, mRNA-2752, **Supplementary Table 1**) (71).

A final approach to prolong tumor-retention is embedding cytokines in depots of co-injected synthetic biomaterials like aluminum hydroxide (alum). Alum aggregates persist for weeks at the site of injection. This has led to a broad use of alum as a safe and effective vaccine adjuvant (2). A recent novel application are cytokine therapeutics that bind to alum deposits via phosphorylated peptide tags (**Figure 1I**) (2). This can restrict cytokine exposure to the injected site and limit cytokine dissemination into circulation. An exciting example is ANK-101, an alum-anchored IL-12 in Phase 1 clinical trials (NCT06171750) (2). The canine surrogate cANK-101 thus far appears safe and tolerable, has shown immune activation and elicited an objective response in a Phase 1 trial in canine melanoma subjects (72). In murine tumor models, alum-bound IL-12 could be detected up to 3 weeks after a single i.t. injection, indicating tumor retention and prolonged exposure (49). Alum-anchoring has also been used preclinically to prolong tumor-retention of i.t.-injected type 1 interferons (52). One theoretical concern is that alum-anchoring might increase the immunogenicity of recombinant cytokines and promote the development of anti-drug antibodies which eventually limit exposure and efficacy. Whether this occurs in patients remains to be shown.

## Conclusions

We consider i.t. administration of cytokines to be more favorable than systemic administration because it can widen the therapeutic index. This is critical to leverage the well-established potency of cytokines as cancer therapeutics, while mitigating their often dose-limiting toxicities, which has prevented a broader utility of cytokines in the clinic.

In our opinion, i.t. delivered cytokines engineered to be retained and stabilized for prolonged periods in tumors are superior therapeutics because they maximize target exposure while minimizing toxic systemic exposure. They also avoid the potential complications of nucleic acids, cells and OVs, particularly related to the control of cytokine exposure and PK. In particular, i.t. injected proteins avoid the liability of excessive and uncontrolled expression of cytokine-encoding nucleic acids or viruses. To achieve optimal tumor retention, bulky moieties such as albumin (**Figure 1F**) or anchoring to synthetic biomaterials (**Figure 1I**) can further improve other retention approaches, including tumor antigen-binding immuno-cytokines or cytokines fused to collagen-binding domains (10, 43, 49, 52, 66). The combination of a collagen-binding moiety with albumin, as realized in CLN-617 (**Figure 1G**), appears particularly powerful because it avoids the need for co-administration of biomaterials such as alum and the theoretical associated risk of eliciting anti-drug antibodies. Nevertheless, both fusion to retention-domains or anchoring to alum combine excellent tumor retention, long PK and high but well controlled tumor exposure with low systemic exposure to achieve high anti-tumor efficacy and safety in preclinical studies. It will be interesting to see how they compare in the clinic.

Because i.t. injection can usually not access all lesions in a patient, ensuring robust abscopal effects is key for success. We believe that this is achievable by combining adaptive and innate immunomodulators with checkpoint blockade. Additionally, properly sequenced combination with T cell engagers, or with antigen-releasing gamma-irradiation or chemotherapy, may be beneficial (10, 53).

For the specific future evolution of i.t. cytokine delivery, we consider co-delivery of synergistic cytokines which activate different arms of adaptive immunity (e.g., IL-2 and IL-12 as in CLN-617), or of cytokines which activate both adaptive and innate immune cells, a particularly promising avenue. We believe that multi-modal molecules containing several cytokines and possibly other immune modulators can facilitate co-delivery. Effective tumor retention will be critical to avoid the increased toxicity potential of cytokine combinations upon systemic exposure. Co-delivery will also require innovative ways to ensure proper exposure of each cytokine to its respective target cells, which may be spatially separated. Finally, different cytokines may act optimally at different times post-delivery and depending on the microenvironment in a given tumor. In one example, alum-bound IFNα and IFNβ had differential efficacies depending on the syngeneic tumor model used (52). These issues may necessitate modifications such as a sequenced delivery or a patient-optimized composition of combination agents for optimal efficacy. Determining the optimal timing and composition of sequenced therapeutics remain considerable challenges, as relevant mechanisms need to be identified and translated into patients, and the required logistics need to be implemented. Without doubt, i.t.-delivered cytokines will provide prospect for innovation for years to come.

## Data Availability

The original contributions presented in the study are included in the article/**Supplementary Material**. Further inquiries can be directed to the corresponding author.

## References

[1] Champiat S Tselikas L Farhane S Raoult T Texier M Lanoy E. Intratumoral immunotherapy: from trial design to clinical practice. Clin Cancer Res. (2021) 27:665–79. doi: 10.1158/1078-0432.CCR-20-0473 32943460

[2] Santollani L Wittrup KD. Spatiotemporally programming cytokine immunotherapies through protein engineering. Immunol Rev. (2023) 320(1):10–28. doi: 10.1111/imr.13234 37409481

[3] Leonard WJ Lin JX. Strategies to therapeutically modulate cytokine action. Nat Rev Drug Discovery. (2023) 22:827–54. doi: 10.1038/s41573-023-00746-x 37542128

[4] Shalhout SZ Miller DM Emerick KS Kaufman HL. Therapy with oncolytic viruses: progress and challenges. Nat Rev Clin Oncol. (2023) 20:160–77. doi: 10.1038/s41571-022-00719-w 36631681

[5] Melero I Castanon E Alvarez M Champiat S Marabelle A. Intratumoural administration and tumour tissue targeting of cancer immunotherapies. Nat Rev Clin Oncol. (2021) 18:558–76. doi: 10.1038/s41571-021-00507-y PMC8130796 34006998

[6] Lazarus HM Ragsdale CE Gale RP Lyman GH. Sargramostim (rhu GM-CSF) as cancer therapy (Systematic review) and an immunomodulator. A drug before its time? Front Immunol. (2021) 12:706186. doi: 10.3389/fimmu.2021.706186 34484202 PMC8416151

[7] Que Y Hu C Wan K Hu P Wang R Luo J. Cytokine release syndrome in COVID-19: a major mechanism of morbidity and mortality. Int Rev Immunol. (2022) 41:217–30. doi: 10.1080/08830185.2021.1884248 PMC7919105 33616462

[8] Zhang JM An J. Cytokines, inflammation, and pain. Int Anesthesiol Clin. (2007) 45:27–37. doi: 10.1097/AIA.0b013e318034194e 17426506 PMC2785020

[9] Krieg C Létourneau S Pantaleo G Boyman O. Improved IL-2 immunotherapy by selective stimulation of IL-2 receptors on lymphocytes and endothelial cells. Proc Natl Acad Sci U S A. (2010) 107:11906–11. doi: 10.1073/pnas.1002569107 PMC2900642 20547866

[10] Wittrup KD Kaufman HL Schmidt MM Irvine DJ. Intratumorally anchored cytokine therapy. Expert Opin Drug Deliv. (2022) 19:725–32. doi: 10.1080/17425247.2022.2084070 PMC9262866 35638290

[11] Rakhit A Yeon MM Ferrante J Fettner S Nadeau R Motzer R. Down-regulation of the pharmacokinetic-pharmacodynamic response to interleukin-12 during long-term administration to patients with renal cell carcinoma and evaluation of the mechanism of this "adaptive response" in mice. Clin Pharmacol Ther. (1999) 65:615–29. doi: 10.1016/S0009-9236(99)90083-8 10391667

[12] Saxton RA Glassman CR Garcia KC. Emerging principles of cytokine pharmacology and therapeutics. Nat Rev Drug Discovery. (2023) 22:21–37. doi: 10.1038/s41573-022-00557-6 36131080 PMC10292932

[13] Holder PG Lim SA Huang CS Sharma P Dagdas YS Bulutoglu B. Engineering interferons and interleukins for cancer immunotherapy. Advanced Drug Delivery Rev. (2022) 182:114112. doi: 10.1016/j.addr.2022.114112 35085624

[14] Atallah-Yunes SA Robertson MJ. Cytokine based immunotherapy for cancer and lymphoma: biology, challenges and future perspectives. Front Immunol. (2022) 13:872010. doi: 10.3389/fimmu.2022.872010 35529882 PMC9067561

[15] Jones DS 2nd Nardozzi JD Sackton KL Ahmad G Christensen E Ringgaard L. Cell surface-tethered IL-12 repolarizes the tumor immune microenvironment to enhance the efficacy of adoptive T cell therapy. Sci Adv. (2022) 8:eabi8075. doi: 10.1126/sciadv.abi8075 35476449 PMC9045725

[16] Hashimoto M Araki K Cardenas MA Li P Jadhav RR Kissick HT. PD-1 combination therapy with IL-2 modifies CD8(+) T cell exhaustion program. Nature. (2022) 610:173–81. doi: 10.1038/s41586-022-05257-0 PMC9793890 36171288

[17] Tang L Zheng Y Melo MB Mabardi L Castano AP Xie YQ. Enhancing T cell therapy through TCR-signaling-responsive nanoparticle drug delivery. Nat Biotechnol. (2018) 36:707–16. doi: 10.1038/nbt.4181 PMC6078803 29985479

[18] Zhang L Morgan RA Beane JD Zheng Z Dudley ME Kassim SH. Tumor-infiltrating lymphocytes genetically engineered with an inducible gene encoding interleukin-12 for the immunotherapy of metastatic melanoma. Clin Cancer Res. (2015) 21:2278–88. doi: 10.1158/1078-0432.CCR-14-2085 PMC4433819 25695689

[19] Nguyen KG Vrabel MR Mantooth SM Hopkins JJ Wagner ES Gabaldon TA. Localized interleukin-12 for cancer immunotherapy. Front Immunol. (2020) 11:575597. doi: 10.3389/fimmu.2020.575597 33178203 PMC7593768

[20] Ferrucci PF Pala L Conforti F Cocorocchio E. Talimogene laherparepvec (T-VEC): an intralesional cancer immunotherapy for advanced melanoma. Cancers (Basel). (2021) 13(6):1383. doi: 10.3390/cancers13061383 33803762 PMC8003308

[21] Zawit M Swami U Awada H Arnouk J Milhem M Zakharia Y. Current status of intralesional agents in treatment of Malignant melanoma. Ann Trans Med. (2021) 9:1038. doi: 10.21037/atm PMC8267328 34277838

[22] CTG. US National Library of Medicine. Rockville Pike, Bethesda, MD: ClinicalTrialsgov (2024).

[23] Wang X Cui C Si L Li C Dai J Mao L. A phase Ib clinical trial of neoadjuvant OrienX010, an oncolytic virus, in combination with toripalimab in patients with resectable stage IIIb to stage IVM1a acral melanoma. J Clin Oncol. (2021) 39:9570–. doi: 10.1200/JCO.2021.39.15_suppl.9570

[24] Cui C Lian B Chi Z Si L Sheng X Li D. OrienX010 oncolytic viral therapy in phase Ic trial of intralesional injection in liver metastases among patients with stage IV melanoma after standard treatment. J Clin Oncol. (2017) 35:e21013–e. doi: 10.1200/JCO.2017.35.15_suppl.e21013

[25] Cui C Wang X Lian B Ji Q Zhou L Chi Z. OrienX010, an oncolytic virus, in patients with unresectable stage IIIC–IV melanoma: a phase Ib study. J ImmunoTherapy Cancer. (2022) 10:e004307. doi: 10.1136/jitc-2021-004307 PMC8984036 35383116

[26] Kundranda MN Rahimian S Shen Y Tan Q Zhao R Esmaeili N. The initial report of phase I trial of VG2025, a non-attenuated HSV-1 oncolytic virus expressing IL-12 and IL-15/RA payloads, in patients with advanced solid tumors. J Clin Oncol. (2023) 41:2580–. doi: 10.1200/JCO.2023.41.16_suppl.2580

[27] Algazi A Bhatia S Agarwala S Molina M Lewis K Faries M. Intratumoral delivery of tavokinogene telseplasmid yields systemic immune responses in metastatic melanoma patients. Ann Oncol. (2020) 31:532–40. doi: 10.1016/j.annonc.2019.12.008 32147213

[28] Algazi AP Twitty CG Tsai KK Le M Pierce R Browning E. Phase II trial of IL-12 plasmid transfection and PD-1 blockade in immunologically quiescent melanoma. Clin Cancer Res. (2020) 26:2827–37. doi: 10.1158/1078-0432.CCR-19-2217 PMC9354653 32376655

[29] Tarhini A Eroglu Z Sarnaik A Zager J Gonzalez R Aquino DD. 617 Neoadjuvant intratumoral TAVO-EP (plasmid IL-12 electro gene transfer) in combination with nivolumab; preliminary clinical and biomarker data in patients with operable locoregionally advanced melanoma. J ImmunoTherapy Cancer. (2022) 10:A649–A. doi: 10.1136/jitc-2022-SITC2022.0617

[30] Danielli R Patuzzo R Di Giacomo AM Gallino G Maurichi A Di Florio A. Intralesional administration of L19-IL2/L19-TNF in stage III or stage IVM1a melanoma patients: results of a phase II study. Cancer Immunol Immunother. (2015) 64:999–1009. doi: 10.1007/s00262-015-1704-6 25971540 PMC11028725

[31] (2023). Nidlegy™ Phase III PIVOTAL trial meets the study’s primary objective demonstrating statistically significant and clinically meaningful improvement in Recurrence-Free Survival for patients with locally advanced fully resectable melanoma [press release]. Online, 10/16/2023.

[32] Hauschild A Hassel JC Ziemer M Rutkowski P Meier FE Flatz L. Phase 3 study (PIVOTAL) of neoadjuvant intralesional daromun vs. immediate surgery in fully resectable melanoma with regional skin and/or nodal metastases. J Clin Oncol. (2024) 42:LBA9501–LBA. doi: 10.1200/JCO.2024.42.17_suppl.LBA9501

[33] Weide B Eigentler TK Pflugfelder A Zelba H Martens A Pawelec G. Intralesional treatment of stage III metastatic melanoma patients with L19–IL2 results in sustained clinical and systemic immunologic responses. Cancer Immunol Res. (2014) 2:668–78. doi: 10.1158/2326-6066.CIR-13-0206 24906352

[34] Albertini MR Yang RK Ranheim EA Hank JA Zuleger CL Weber S. Pilot trial of the hu14.18-IL2 immunocytokine in patients with completely resectable recurrent stage III or stage IV melanoma. Cancer Immunol Immunother. (2018) 67:1647–58. doi: 10.1007/s00262-018-2223-z PMC6168354 30073390

[35] Albertini MR Morris ZS Hank JA Ranheim E Zuleger CL McDowell K. Phase I/II trial of intratumoral administration of hu14.18-IL2, with local radiation, nivolumab, and ipilimumab in subjects with advanced melanoma. J Clin Oncol. (2021) 39:TPS9591–TPS. doi: 10.1200/JCO.2021.39.15_suppl.TPS9591

[36] Yang RK Kuznetsov IB Ranheim EA Wei JS Sindiri S Gryder BE. Outcome-related signatures identified by whole transcriptome sequencing of resectable stage III/IV melanoma evaluated after starting Hu14.18-IL2. Clin Cancer Res. (2020) 26:3296–306. doi: 10.1158/1078-0432.CCR-19-3294 PMC7334053 32152202

[37] Raeber ME Sahin D Karakus U Boyman O. A systematic review of interleukin-2-based immunotherapies in clinical trials for cancer and autoimmune diseases. EBioMedicine. (2023) 90:104539. doi: 10.1016/j.ebiom.2023.104539 37004361 PMC10111960

[38] Weide B Eigentler TK Pflugfelder A Leiter U Meier F Bauer J. Survival after intratumoral interleukin-2 treatment of 72 melanoma patients and response upon the first chemotherapy during follow-up. Cancer Immunology Immunother. (2011) 60:487–93. doi: 10.1007/s00262-010-0957-3 PMC11029697 21174093

[39] Boyd KU Wehrli BM Temple CLF. Intra-lesional interleukin-2 for the treatment of in-transit melanoma. J Surg Oncol. (2011) 104:711–7. doi: 10.1002/jso.21968 21744347

[40] von Wussow P Block B Hartmann F Deicher H. Intralesional interferon-alpha therapy in advanced Malignant melanoma. Cancer. (1988) 61:1071–4. doi: 10.1002/(ISSN)1097-0142 3342367

[41] Robinson WA Mughal TI Thomas MR Johnson M Spiegel RJ. Treatment of metastatic Malignant melanoma with recombinant interferon alpha 2. Immunobiology. (1986) 172:275–82. doi: 10.1016/S0171-2985(86)80109-7 3804370

[42] Sertoli MR Bernengo MG Ardizzoni A Brunetti I Falcone A Vidili MG. Phase II trial of recombinant alpha-2b interferon in the treatment of metastatic skin melanoma. Oncology. (1989) 46:96–8. doi: 10.1159/000226693 2710482

[43] Mehta NK Rakhra K Meetze KA Li B Momin N Chang JY. CLN-617 retains IL-2 and IL-12 in injected tumors to drive robust and systemic immune-mediated antitumor activity. Cancer Immunol Res. (2024) 12(8):1022–38. doi: 10.1158/2326-6066.c.7380038.v1 PMC11292205 38842347

[44] Mehta NK Rakhra K Meetze K Wittrup KD Michaelson JS Baeuerle PA. Abstract 1839: CLN-617 is a first-in-class fusion protein that retains IL-2 and IL-12 in the injected tumor and potently triggers systemic anti-tumor immunity. Cancer Res. (2023) 83:1839–. doi: 10.1158/1538-7445.AM2023-1839

[45] Press releases. Available online at: https://ankyratx.com/2023. (Accessed January 31, 2024).

[46] Yuan J Khilnani A Brody J Andtbacka RHI Hu-Lieskovan S Luke JJ. Current strategies for intratumoural immunotherapy – Beyond immune checkpoint inhibition. Eur J Cancer. (2021) 157:493–510. doi: 10.1016/j.ejca.2021.08.004 34561127

[47] Xu W Atkinson VG Menzies AM. Intratumoural immunotherapies in oncology. Eur J Cancer. (2020) 127:1–11. doi: 10.1016/j.ejca.2019.12.007 31962197

[48] Humeau J Le Naour J Galluzzi L Kroemer G Pol JG. Trial watch: intratumoral immunotherapy. Oncoimmunology. (2021) 10:1984677. doi: 10.1080/2162402X.2021.1984677 34676147 PMC8526014

[49] Agarwal Y Milling LE Chang JYH Santollani L Sheen A Lutz EA. Intratumourally injected alum-tethered cytokines elicit potent and safer local and systemic anticancer immunity. Nat BioMed Eng. (2022) 6:129–43. doi: 10.1038/s41551-021-00831-9 PMC9681025 35013574

[50] Yun C-O Hong J Yoon A-R. Current clinical landscape of oncolytic viruses as novel cancer immunotherapeutic and recent preclinical advancements. Front Immunol. (2022) 13. doi: 10.3389/fimmu.2022.953410 PMC9458317 36091031

[51] Lewis ND Sia CL Kirwin K Haupt S Mahimkar G Zi T. Exosome surface display of IL12 results in tumor-retained pharmacology with superior potency and limited systemic exposure compared with recombinant IL12. Mol Cancer Ther. (2021) 20:523–34. doi: 10.1158/1535-7163.MCT-20-0484 33443094

[52] Lutz EA Agarwal Y Momin N Cowles SC Palmeri JR Duong E. Alum-anchored intratumoral retention improves the tolerability and antitumor efficacy of type I interferon therapies. Proc Natl Acad Sci U S A. (2022) 119:e2205983119. doi: 10.1073/pnas.2205983119 36037341 PMC9457244

[53] Nabrinsky E Macklis J Bitran J. A review of the abscopal effect in the era of immunotherapy. Cureus. (2022) 14:e29620. doi: 10.7759/cureus.29620 36321062 PMC9604762

[54] Atkins MB Lotze MT Dutcher JP Fisher RI Weiss G Margolin K. High-dose recombinant interleukin 2 therapy for patients with metastatic melanoma: analysis of 270 patients treated between 1985 and 1993. J Clin Oncol. (1999) 17:2105–16. doi: 10.1200/JCO.1999.17.7.2105 10561265

[55] Ahmadzadeh M Rosenberg SA. IL-2 administration increases CD4+ CD25(hi) Foxp3+ regulatory T cells in cancer patients. Blood. (2006) 107:2409–14. doi: 10.1182/blood-2005-06-2399 PMC1473973 16304057

[56] Radny P Caroli UM Bauer J Paul T Schlegel C Eigentler TK. Phase II trial of intralesional therapy with interleukin-2 in soft-tissue melanoma metastases. BrJCancer. (2003) 89:1620–6. doi: 10.1038/sj.bjc.6601320 PMC2394422 14583759

[57] Dehesa LA Vilar-Alejo J Valerón-Almazán P Carretero G. [Experience in the treatment of cutaneous in-transit melanoma metastases and satellitosis with intralesional interleukin-2]. Actas Dermosifiliogr. (2009) 100:571–85. doi: 10.1016/S0001-7310(09)71905-2 19715642

[58] Albertini MR Hank JA Gadbaw B Kostlevy J Haldeman J Schalch H. Phase II trial of hu14.18-IL2 for patients with metastatic melanoma. Cancer Immunol Immunother. (2012) 61:2261–71. doi: 10.1007/s00262-012-1286-5 PMC3502633 22678096

[59] Shusterman S London WB Gillies SD Hank JA Voss SD Seeger RC. Antitumor activity of hu14.18-IL2 in patients with relapsed/refractory neuroblastoma: a Children's Oncology Group (COG) phase II study. J Clin Oncol. (2010) 28:4969–75. doi: 10.1200/JCO.2009.27.8861 PMC3020698 20921469

[60] Kali A. TNFerade, an innovative cancer immunotherapeutic. Indian J Pharmacol. (2015) 47:479–83. doi: 10.4103/0253-7613.165190 PMC4621666 26600634

[61] Bechter O Loquai C Champiat S Baurain JF Grob J-J Utikal J. Abstract LB198: A first-in-human, open-label, multicenter study of intratumoral SAR441000 (mixture of cytokine encoding mRNAs), as monotherapy and in combination with cemiplimab in patients with advanced solid tumors. Cancer Res. (2023) 83:LB198–LB. doi: 10.1158/1538-7445.AM2023-LB198

[62] Bechter O Utikal J Baurain J-F Massard C Sahin U Derhovanessian E. 391 A first-in-human study of intratumoral SAR441000, an mRNA mixture encoding IL-12sc, interferon alpha2b, GM-CSF and IL-15sushi as monotherapy and in combination with cemiplimab in advanced solid tumors. J ImmunoTherapy Cancer. (2020) 8:A237–A8. doi: 10.1136/jitc-2020-SITC2020.0391

[63] Schloesser P. Sanofi, BioNTech cut early-stage mRNA cancer therapy from pipeline: ENDPOINTS NEWS(2023). Available online at: https://endpts.com/sanofi-biontech-terminate-development-of-mrna-cancer-candidate/. (Accessed July 28, 2023).

[64] van Herpen CM Looman M Zonneveld M Scharenborg N de Wilde PC van de Locht L. Intratumoral administration of recombinant human interleukin 12 in head and neck squamous cell carcinoma patients elicits a T-helper 1 profile in the locoregional lymph nodes. Clin Cancer Res. (2004) 10:2626–35. doi: 10.1158/1078-0432.CCR-03-0304 15102664

[65] Eton O Rosenblum MG Legha SS Zhang W Jo East M Bedikian A. Phase I trial of subcutaneous recombinant human interleukin-2 in patients with metastatic melanoma. Cancer. (2002) 95:127–34. doi: 10.1002/cncr.10631 12115326

[66] Momin N Mehta NK Bennett NR Ma L Palmeri JR Chinn MM. Anchoring of intratumorally administered cytokines to collagen safely potentiates systemic cancer immunotherapy. Sci Trans Med. (2019) 11:eaaw2614. doi: 10.1126/scitranslmed.aaw2614 PMC7811803 31243150

[67] Momin N Palmeri JR Lutz EA Jailkhani N Mak H Tabet A. Maximizing response to intratumoral immunotherapy in mice by tuning local retention. Nat Commun. (2022) 13:109. doi: 10.1038/s41467-021-27390-6 35013154 PMC8748612

[68] Stinson JA Barbosa MMP Sheen A Momin N Fink E Hampel J. Tumor-localized interleukin-2 and interleukin-12 combine with radiation therapy to safely potentiate regression of advanced Malignant melanoma in pet dogs. Clin Cancer Res. (2024). doi: 10.1158/1078-0432.CCR-24-0861 PMC11398984 38980919

[69] Lebbink RJ van den Berg MC de Ruiter T Raynal N van Roon JA Lenting PJ. The soluble leukocyte-associated Ig-like receptor (LAIR)-2 antagonizes the collagen/LAIR-1 inhibitory immune interaction. J Immunol. (2008) 180:1662–9. doi: 10.4049/jimmunol.180.3.1662 18209062

[70] Ramos MIP Tian L de Ruiter EJ Song C Paucarmayta A Singh A. Cancer immunotherapy by NC410, a LAIR-2 Fc protein blocking human LAIR-collagen interaction. eLife. (2021) 10:e62927. doi: 10.7554/eLife.62927.sa2 34121658 PMC8225389

[71] Patel M Jimeno A Wang D Stemmer S Bauer T Sweis R. 539 Phase 1 study of mRNA-2752, a lipid nanoparticle encapsulating mRNAs encoding human OX40L/IL-23/IL-36γ, for intratumoral (ITu) injection +/- durvalumab in advanced solid tumors and lymphoma. J ImmunoTherapy Cancer. (2021) 9:A569–A. doi: 10.1136/jitc-2021-SITC2021.539

[72] Barbosa MMP Lopez AJ Uyehara R Kamerer RL Schmidt M Battula S. Abstract 6347: Preliminary results of an exploratory phase I clinical trial of anchored canine interleukin-12 (cANK-101) in dogs with advanced oral Malignant melanoma. Cancer Res. (2023) 83:6347–. doi: 10.1158/1538-7445.AM2023-6347

