# Supplementary material for: Intratumoral injection and retention hold promise to improve cytokine therapies for cancer
Source: Front Oncol. 2024 Aug 26;14:1456658. doi: 10.3389/fonc.2024.1456658 (PMC11381304; doi:10.3389/fonc.2024.1456658)
Supplement: Supplementary file 1 [file Table1.docx]

Supplementary Material

# Supplementary Table 1

**I.t. injected cytokines that have reached clinical trials, continued**

| **Modality** | **Asset** | **Cytokine(s)** | **Phase** | **Indication(s)** | **Comments*** | **References** |
| --- | --- | --- | --- | --- | --- | --- |
| OV | MEDI9253 | IL-12 | - 1 | Solid tumors (MSS) | - *Discontinued* - Durvalumab combo | (58, 73) |
|  | Ad-RTS-hIL-12 (Precigen /Alaunos/  Ziopharm) | IL-12, drug-inducible | - 1/2 | GBM, HR+ BC, melanoma | - *All trials completed or terminated* - Limited single-agent activity (12.7 months mOS) and combination efficacy - Well tolerated - Variable increases of IL-12 and IFNγ in blood | (58, 74, 75) |
|  | Ad5-yCD/ *mut*TK_SR39_ *rep*-hIL-12 (Henry Ford Health System) | IL-12 (+ Chemo) | - 1 | PDAC, prostate cancer | - 18.1 months mOS (PDAC) - Elevated serum IL-12, IFNγ and CXCL10 in ≥42% of patients - Viral DNA detected in serum of some patients - MTD not reached | (58, 76) |
|  | MVR-T3011-IT (ImmVira) | IL-12 (+ αPD-1) | - 1/2 | Melanoma, various solid tumors | - Patients with advanced melanoma or HNSCC showed ORR of 21.1-25% - Other various solid tumors showed ORR of ~11% - Well tolerated - Viral DNA undetected in blood, urine, saliva in >80% of patients - Increased CD8 TIL | (58, 77, 78) |
|  | ASP9801 (Astellas) | IL-12 + IL-7 | - 1 | Solid Tumors | - *Discontinued* | (58) |
|  | AZD4820 (Transgene/AstraZeneca) | IL-12 | - 1 | Solid tumors | - *Terminated* | (79) |
|  | ONCR-177 (Oncorus) | IL-12, CCL4, FLT3L, PD-  1, CTLA-4 | - 1 | Solid Tumors | - *Discontinued* - No objective responses - No DLT | (58, 80), Oncorus press release 2022 (company closed) |
|  | TBio-6517/ RIVAL-01/ TAK-605  (Turnstone Biologics) | IL-12, CTLA-4, FLT3L (± αPD-1) | - 1/2 | Solid Tumors | - *Terminated, Takeda returned rights* | (58, 81) |
|  | Ad-TD-nsIL12 (Capital Medical University) | non-secretory IL-12 | - 1 | Glioma | - Non-secretory IL-12 designed to limit systemic exposure | (58, 82) |
|  | Igrelimogene litadenorepvec  (Ad5/3-E2F-d24-hTNF-IRES-hIL2; TILT-123) (Tilt Biotherapeutics) | IL-2 + TNFα (± αPD-1) | - 1 | Solid tumors, melanoma, OC | - - PROTA (NCT05271318): - - Combination with Pembrolizumab in r/r OC - - DLT not observed - - 64.3% SD - - mPFS 105 days - - mOS 280 days | (58, 83) |
|  | CVD-1301.V01 (Hangzhou Converd) | IL-21 | - 1 | Solid tumors | - *Clinical study initiated 2023* | (58) |
|  | RP1 (Replimune) | GM-CSF | - 1/2 | Skin cancers | - Melanoma: 37.4% ORR + Nivolumab - Well tolerated | (58, 84) |
|  | RP2 (Replimune) | GM-CSF, CTLA-4 | - 1/2 | Solid tumors, CRC | - Uveal Melanoma: 28.6% ORR + CPI - Favorable safety profile | (58, 85) |
|  | BT-001 (Transgene/BioInvent) | GM-CSF, CTLA-4 | - 1/2 | Solid tumors |  | (58) |
|  | ONCOS-102 (Oncos) | GM-CSF | - 1/2 | Melanoma, mesothelioma | - Mesothelioma: + P/C, trend for prolonged OS only 1L - Melanoma: 35% ORR + CPI - Well tolerated | (58, 86, 87) |
|  | OH2 (Wuhan Binhui Biotechnology) | GM-CSF (±αPD-1 or Chemo) | - 1/2 | Solid tumors, CRC r, melanoma, bladder cancer, pancreatic cancer | - In solid tumors, 5% immune PR as monotherapy, 14% in combo - In stage IIIC-IV melanoma, 25% ORR, mOS ≥20.8 months (low n). - Plus pucotenlimab/ radiotherapy, 40% ORR. - Well tolerated | (58, 88-91) |
|  | Pexa-Vec/JX-594 (Pexastimogene devacirepvec, SillaJen) | GM-CSF (±αPD-1 or αCTLA-4 or MKI) | - 1-3 | Solid tumors | - *Most completed* - HCC, monotherapy: mOS >423 days, 3% SAE - HCC, + Nivolumab, 33.3% ORR. - HCC, + Sorafenib, 19.2% ORR similar to Sorafenib alone (20.9%) - HCC, + Nivolumab, 85.7% SAE. + Sorafenib, 53.67% SAE exceeds Sorafenib alone - Pexa-Vec did not improve OS as second-line therapy after sorafenib failure in HCC - Little efficacy in pediatric patients | (5, 58, 92-94) |
|  | SynOV1.1 (Beijing Syngentech) | GM-CSF | - 1 | Solid Tumors |  | (58) |
|  | MEM-288 (Memgen) | IFNβ (+ CD40L) | - 1 | Solid tumors, NSCLC | - 30% SD - No DLT - Systemic IFN response | (58, 95) |
|  | VSV-IFNbetaTYRP1 (Mayo Clinic) | IFNβ (+ TYRP1) | - 1 | Melanoma | - *- No data* | (58) |
|  | Voyager-V1/VV1/VSV-IFNβ-NIS (Vyriad) | IFNβ (±αPD-(L)1 or αCTLA-4) | - 1 | Solid tumors, HCC, CRC, HNSCC, melanoma | - Completed  - No responses beyond SD   - - Well tolerated | (58, 96-98) |
| Adenovirus | TNFerade (Golnerminogene pradenovec/Ad GV.EGR.TNF.11D, GenVec) | TNFα (+ 5-FU/radiation) | - 1-3 | Solid tumors, pancreatic Cancer | - TNFα induced by chemoradiation - *Phase 3 study in pancreatic cancer discontinued for lack of efficacy vs SOC* - Well tolerated | (42, 58, 99) |
| Vaccine, viral | KB707 (Krystal Bio) | IL-2 + IL-12 | - 1 | Solid tumors | *Clinical study Initiated 2023* | (58) |
|  | GEN2 (GenVivo) | GM-CSF, HSV-TK | - 1 | Liver tumors |  | (58) |
|  | VV-GMCSFLact (Oncostar) | GM-CSF, lactaptin | - 1 | Breast cancer |  | (58) |
| cDNA Plasmid | phIL12 (Institute of Oncology Ljubljana) | IL-12 | - 1 | Basal cell carcinoma in head and neck region |  | (58, 100) |
| mRNA | MEDI1191 (Astra Zeneca/  Moderna) | IL-12 | - 1 (+ Durvalumab) | Solid tumors | - *Discontinued* - Low ORR of 8.2% - Low induction of systemic IL-12 & IFNγ - Some abscopal effects - Tolerable | (58, 101, 102) |
|  | ABOD2011 (Chinese Academy of Medical Sciences) | IL-12 | - 1 | Solid tumors |  | (58) |
|  | BNT131/SAR441000 (BioNTech/  Sanofi) | IL-12, IFNα, GM-CSF + IL-15 sushi domain | - 1 (± Cemiplimab) | Solid Tumors | - *Discontinued* - In melanoma, 5% ORR - Well tolerated - Limited abscopal effects - Some systemic immune responses | (43-45, 58) |
| mRNA LNP | JCXH-211 (Immorna Biotherapeutics) | IL-12 | - 1 | Solid tumors, GBM | - *NCT05727839 Ph 1:* - Tumor shrinkage including abscopal effects - Increased T and NK cell infiltration in lesions - No DLT - No drug-related SAE | (58, 103) |
|  | mRNA-2752 (Moderna) | IL-23, IL-36, OX40 (+αPD-(L)1) | - 1 | Solid tumors, lymphoma, high-risk ductal carcinoma in situ (DCIS) | - No responses as monotherapy - 6.7% ORR + Durvalumab - Safe and tolerable (1 DLT + Durvalumab) | (56, 58) |
|  | STX-001 (Strand Therapeutics) | IL-12 | - 1 (± Pembrolizumab) | Solid tumors |  | (58) |
| MSC vaccine | GX-051 (Genexine) | IL-12 | - 1 | HNSCC | - *Unknown status* - Human IL-12 secreting MSC | (58) |
| Cytokine-decorated exosomes | CDK-003/ExoIL-12 (Codiak) | IL-12 | - 1 | CTCL | - *Terminated* - Evidence of efficacy and abscopal effects (n=2) - Favorable safety - No detectable systemic IL-12 exposure | (58), CODIAK press release, 6/20/2022 (company filed for bankruptcy) |
| Recombinant or natural cytokine | Proleukin | IL-2 |  |  | - *Case study* - Increased tumor necrosis - Well tolerated | (104) |
|  | IL-2 | IL-2 (+ αPD-1 + RT) | - 1/2 | PD-1 refractory solid tumors |  | (58) |
|  |  | IL-2 (+αCTLA-4) | - 2 | Melanoma | - *Completed* | (58) |
|  |  | GM-CSF | - 1 | Melanoma | - Partial regressions of injected and/or uninjected metastases in 3/13 patients - Increased CD4 TIL and Langerhans cells | (105) |
|  | Recombinant IFNα 2b | IFNα + IL-2 |  | Cystic GBM | - No efficacy - No side effects | (106) |
|  | Natural human IFNβ | IFNβ |  | Melanoma | - No objective responses (n=3) - Tumor cell apoptosis - Lymphocyte infiltration | (107) |

*Monotherapy unless indicated otherwise. Abbreviations: Chemo, chemotherapy; CPI, checkpoint inhibitor/blocker; CTCL, cutaneous T-cell lymphoma; CTG, ClinicalTrials.gov; DLT, dose-limiting toxicity; GBM, glioblastoma; HR, hazard ratio; MKI, multi-kinase inhibitor; mPFS, median progression-free survival; mOS, median overall survival; MSC, mesenchymal stem cell; MSS, microsatellite-stable; MTD, maximum tolerated dose; NMIBC , Non-Muscle Invasive Bladder Cancer; OC, ovarian cancer; ORR, overall response rate; OS, overall survival; mOS, median OS; P/C, pemetrexed/cisplatin; Pembro, pembrolizumab; RT, radiation therapy; SAE, serious adverse events; SD, stable disease; SOC, standard of care; TRAE, treatment-related adverse effects.

# Supplementary References

73. Purroy N, Durham N, Phillips M, Hattersley MM, Jung L, Davar D, et al. Abstract CT218: First-in-human trial of intravenous MEDI9253, an oncolytic virus, in combination with durvalumab in patients with advanced solid tumors. Cancer Res. 2022;82(12_Supplement):CT218-CT.

74. Chiocca EA, Yu JS, Lukas RV, Solomon IH, Ligon KL, Nakashima H, et al. Regulatable interleukin-12 gene therapy in patients with recurrent high-grade glioma: Results of a phase 1 trial. Sci Transl Med. 2019;11(505).

75. Lebel FM, Barrett JA, McArthur HL, Buck JY, Demars N, Mackenna R, et al. Demonstration of anti-tumor immunity via intratumoral regulated platform ad-RTS-hIL-12 in advanced breast cancer and recurrent glioblastoma patients. Journal of Clinical Oncology. 2018;36(15_suppl):3038-.

76. Barton KN, Siddiqui F, Pompa R, Freytag SO, Khan G, Dobrosotskaya I, et al. Phase I trial of oncolytic adenovirus-mediated cytotoxic and interleukin-12 gene therapy for the treatment of metastatic pancreatic cancer. Mol Ther Oncolytics. 2021;20:94-104.

77. Ji D, Weitao Y, Tong X, Zhang C, Wang F, Chen Z, et al. A phase 1/2a study of T3011, an oncolytic HSV expressing IL-12 and PD-1 antibody, administered via intratumoral (IT) injection as monotherapy in advanced solid tumors. Journal of Clinical Oncology. 2023;41(16_suppl):2520-.

78. Press Releases [press release]. <https://www.immviragroup.com/>: <https://www.immviragroup.com/2023>.

79. Taylor N. AstraZeneca scraps pact with Riva-led Transgene in further blow to oncolytic viruses 2023 [Available from: <https://www.fiercebiotech.com/biotech/astrazeneca-scraps-pact-riva-led-transgene-further-blow-oncolytic-viruses>.

80. Park JC, Soliman H, Falchook G, Owonikoko T, Spreafico A, Massarelli E, et al. 511 Initial results of a phase 1 study of intratumoral ONCR-177, an oncolytic herpes-simplex virus-1 expressing five immunomodulatory transgenes, in subjects with advanced injectable tumors. Journal for ImmunoTherapy of Cancer. 2021;9(Suppl 2):A542-A.

81. Taylor N. Takeda cuts ties to cancer drug landed in $120M Turnstone deal: Fierce Biotech; 2022 [Available from: <https://www.fiercebiotech.com/biotech/takeda-cuts-ties-cancer-drug-landed-120m-turnstone-deal-part-pipeline-clear-out>.

82. Zhang Z, Zhang C, Miao J, Wang Z, Wang Z, Cheng Z, et al. A Tumor-Targeted Replicating Oncolytic Adenovirus Ad-TD-nsIL12 as a Promising Therapeutic Agent for Human Esophageal Squamous Cell Carcinoma. Cells. 2020;9(11).

83. Santos JM, Block MS, Maenpaa JU, Clubb J, Alanko T, Choong GMY, et al. PROTA: A phase I clinical trial combining an oncolytic adenovirus encoding for TNFa and IL-2 with pembrolizumab for the treatment of platinum-resistant or -refractory ovarian cancer. Journal of Clinical Oncology. 2024;42(16_suppl):5562-.

84. Chmielowski B, Milhem MM, Sacco JJ, Bowles TL, Tsai KK, In GK, et al. Initial efficacy and safety of RP1 + nivolumab in patients with anti–PD-1–failed melanoma from the ongoing phase 1/2 IGNYTE study. Journal of Clinical Oncology. 2023;41(16_suppl):9509-.

85. Sacco JJ, Harrington KJ, Olsson-Brown A, Chan TY, Nenclares P, Leslie I, et al. Preliminary safety and efficacy results from an open-label, multicenter, phase 1 study of RP2 as a single agent and in combination with nivolumab in a cohort of patients with uveal melanoma. Journal of Clinical Oncology. 2023;41(16_suppl):9527-.

86. Hansen TB, Cedres Perez S, Ricordel C, Levitsky V, Ottesen LH, Paz-Ares LG. Granular analysis of individual immune-related gene expression in a randomized phase I/II study of the oncolytic adenovirus, ONCOS-102, in combination with pemetrexed/cisplatin (P/C) in patients (pts) with unresectable malignant pleural mesothelioma (MPM). Journal of Clinical Oncology. 2023;41(16_suppl):e20536-e.

87. Shoushtari AN, Olszanski AJ, Nyakas M, Hornyak TJ, Wolchok JD, Levitsky V, et al. Pilot Study of ONCOS-102 and Pembrolizumab: Remodeling of the Tumor Microenvironment and Clinical Outcomes in Anti-PD-1-Resistant Advanced Melanoma. Clin Cancer Res. 2023;29(1):100-9.

88. Zhang B, Huang J, Tang J, Hu S, Luo S, Luo Z, et al. Intratumoral OH2, an oncolytic herpes simplex virus 2, in patients with advanced solid tumors: a multicenter, phase I/II clinical trial. Journal for ImmunoTherapy of Cancer. 2021;9(4):e002224.

89. Wang X, Cui C, Lian B, Si L, Chi Z, Sheng X, et al. A phase Ia/Ib study evaluating the safety and efficacy of intratumorally administrated OH2, an oncolytic herpes simplex virus 2, in unresected stage IIIC to IV melanoma patients. Journal of Clinical Oncology. 2022;40(16_suppl):e21537-e.

90. Huang J, Zhang B, Tang J, Chang Q, Zhang R, Geng C, et al. Safety and tolerability of intratumorally administered OH2, an oncolytic herpes simplex virus 2, in patients with advanced solid tumors: A phase I dose escalation clinical study. Journal of Clinical Oncology. 2020;38(15_suppl):3139-.

91. Cui C, Wang X, Wang H, Yin S, Cong Y, Lian B, et al. The phase 1 clinical trial of anti–PD-1 (pucotenlimab) plus intrahepatic injection of oncolytic virus (OH2) combined with radiotherapy of liver metastasis in stage IV melanoma. Journal of Clinical Oncology. 2023;41(16_suppl):e21545-e.

92. Moehler M, Heo J, Lee HC, Tak WY, Chao Y, Paik SW, et al. Vaccinia-based oncolytic immunotherapy Pexastimogene Devacirepvec in patients with advanced hepatocellular carcinoma after sorafenib failure: a randomized multicenter Phase IIb trial (TRAVERSE). Oncoimmunology. 2019;8(8):1615817.

93. Cripe TP, Ngo MC, Geller JI, Louis CU, Currier MA, Racadio JM, et al. Phase 1 study of intratumoral Pexa-Vec (JX-594), an oncolytic and immunotherapeutic vaccinia virus, in pediatric cancer patients. Mol Ther. 2015;23(3):602-8.

94. Breitbach CJ, Bell JC, Hwang TH, Kirn DH, Burke J. The emerging therapeutic potential of the oncolytic immunotherapeutic Pexa-Vec (JX-594). Oncolytic Virother. 2015;4:25-31.

95. Saltos AN, Arrowood C, Beasley G, Ronald J, El-Haddad G, Guerra-Guevara L, et al. A phase 1 first-in-human study of interferon beta (IFNβ) and membrane-stable CD40L expressing oncolytic virus (MEM-288) in solid tumors including non–small-cell lung cancer (NSCLC). Journal of Clinical Oncology. 2023;41(16_suppl):2569-.

96. Powell SF, Patel MR, Merchan J, Strauss J, Cripe TP, Old MO, et al. VSV-IFN&#x3b2;-NIS intratumoral (IT) injection: A first-in-human (FIH), phase I study of an innovative oncolytic virotherapy, alone and with an anti-PD-L1 antibody, in patients with refractory solid tumors. Annals of Oncology. 2018;29:viii439.

97. Sznol M, Lutzky J, Adjei AA, Powell SF, He AR, Patel M, et al. Phase II trial of Voyager-V1 (vesicular stomatitis virus expressing human IFNβ and NIS, VV1), in combination with cemiplimab (C) in patients with NSCLC, melanoma, HCC or endometrial carcinoma. Journal of Clinical Oncology. 2020;38(15_suppl):TPS3161-TPS.

98. Ram P, Tung N, Sharma H, Fulton B, Peng K-W, Russell S, et al. 851 Voyager V1 (VV1) oncolytic virus combined with immune checkpoint therapy boosts CTL responses to multiple tumor antigens and correspondingly deepens tumor responses in murine models of melanoma, lung and colon cancer. Journal for ImmunoTherapy of Cancer. 2022;10(Suppl 2):A890-A.

99. Herman JM, Wild AT, Wang H, Tran PT, Chang KJ, Taylor GE, et al. Randomized phase III multi-institutional study of TNFerade biologic with fluorouracil and radiotherapy for locally advanced pancreatic cancer: final results. J Clin Oncol. 2013;31(7):886-94.

100. Groselj A, Bosnjak M, Jesenko T, Cemazar M, Markelc B, Strojan P, Sersa G. Treatment of skin tumors with intratumoral interleukin 12 gene electrotransfer in the head and neck region: a first-in-human clinical trial protocol. Radiol Oncol. 2022;56(3):398-408.

101. Castañón E, Zamarin D, Carneiro BA, Marron T, Patel SP, Subbiah V, et al. Abstract CT004: Intratumoral (IT) MEDI1191 + durvalumab (D): Update on the first-in-human study in advanced solid tumors. Cancer Res. 2023;83(8_Supplement):CT004-CT.

102. Abadier M, Jennings E, Eyles J, Martin P, Pilataxi F, Wu Y, et al. 708 MEDI1191 (IL-12 mRNA) induces peripheral and intratumoral immunostimulatory effect in patients with cutaneous or subcutaneous (C/SC) lesions. Journal for ImmunoTherapy of Cancer. 2022;10(Suppl 2):A741-A.

103. Le NT. A phase I study to evaluate the safety and tolerability of JCXH-211 (a self-replicating mRNA encoding IL-12) intratumoral injection in patients with malignant solid tumors: Results from the phase Ia dose escalation. Journal of Clinical Oncology. 2024;42(16_suppl):2539-.

104. Gutwald JGJ, Groth W, Mahrle G. Perilumoral injections of interleukin 2 induce tumour regression in metastatic malignant melanoma. British Journal of Dermatology. 1994;130(4):541-2.

105. Si Z, Hersey P, Coates AS. Clinical responses and lymphoid infiltrates in metastatic melanoma following treatment with intralesional GM-CSF. Melanoma Res. 1996;6(3):247-55.

106. Vaquero J, Martínez R. Intratumoral immunotherapy with interferon-alpha and interleukin-2 in glioblastoma. Neuroreport. 1992;3(11):981-3.

107. Kubo H, Ashida A, Matsumoto K, Kageshita T, Yamamoto A, Saida T. Interferon-β therapy for malignant melanoma: the dose is crucial for inhibition of proliferation and induction of apoptosis of melanoma cells. Archives of Dermatological Research. 2008;300(6):297-301.
